# A Deep Learning Approach for Classifying Benign, Malignant, and Borderline Ovarian Tumors Using Convolutional Neural Networks and Generative Adversarial Networks

**DOI:** 10.3390/medsci14010089

**Published:** 2026-02-14

**Authors:** Maria Giourga, Ioannis Petropoulos, Sofoklis Stavros, Anastasios Potiris, Kallirroi Goula, Efthalia Moustakli, Anthi-Maria Papahliou, Maria-Anastasia Daskalaki, Margarita Segou, Alexandros Rodolakis, George Daskalakis, Ekaterini Domali

**Affiliations:** 1 First Department of Obstetrics and Gynecology, Alexandra Hospital, Medical School, National and Kapodistrian University of Athens, 11528 Athens, Greece; 2 School of Electrical & Computer Engineering, National Technical University of Athens, 15772 Athens, Greece; 3 Third Department of Obstetrics and Gynecology, University General Hospital “ATTIKON”, Medical School, National and Kapodistrian University of Athens, 12462 Athens, Greece; sfstavrou@med.uoa.gr (S.S.); apotiris@med.uoa.gr (A.P.); 4 Department of Pathology, Alexandra Hospital, Medical School, National and Kapodistrian University of Athens, 11528 Athens, Greece; 5 Department of Nursing, School of Health Sciences, University of Ioannina, 45500 Ioannina, Greece; 6 British Geological Survey, The Lyell Centre, Research Avenue South, Edinburgh EH14 4AP, UK

**Keywords:** ovarian mass, ovarian cancer, ultrasound imaging, artificial intelligence, deep convolutional generative adversarial network (DCGAN), deep learning

## Abstract

**Background/Objectives**: Accurate preoperative characterization of ovarian masses is essential for appropriate clinical management, particularly for borderline ovarian tumors (BOTs), which are less common and often difficult to distinguish from benign or malignant lesions on ultrasound. Although expert subjective ultrasound assessment achieves high diagnostic accuracy, limited availability of highly trained sonologists restricts its widespread application. Artificial intelligence-based approaches offer a potential solution; however, the low prevalence of BOTs restricts the development of robust deep learning models due to severe class imbalance. This study aimed to develop a Convolutional Neural Network (CNN)-based classifier enhanced with Generative Adversarial Networks (GANs) to improve the discrimination of ovarian masses as benign, malignant, or BOT using ultrasound images. **Methods**: A total of 3816 ultrasound images from 636 ovarian masses were retrospectively analyzed, including 390 benign lesions, 202 malignant tumors, and 44 BOTs. To address class imbalance, a Deep Convolutional GAN (DCGAN) was used to generate 2000 synthetic BOT images for data augmentation. A three-class ensemble CNN model integrating VGG16, ResNet50, and InceptionNetV3 architectures was developed. Performance was assessed on an independent test set and compared with a baseline model trained without DCGAN augmentation. **Results**: The incorporation of DCGAN-generated BOT images significantly enhanced classification performance. The BOT F1-score increased from 68.4% to 86.5%, while overall accuracy improved from 84.7% to 91.5%. For BOT identification, the final model achieved a sensitivity of 88.2% and specificity of 85.1%. Class-specific AUCs were 0.96 for benign lesions, 0.94 for malignant tumors, and 0.91 for BOTs. **Conclusions**: DCGAN-based augmentation effectively expands limited ultrasound datasets and improves CNN performance, particularly for BOT detection. This approach demonstrates potential as a decision support tool for preoperative assessment of ovarian masses.

## 1. Introduction

Ovarian cancer remains one of the most lethal gynecologic malignancies, with high mortality rates due to the lack of effective screening methods and delayed diagnosis caused by late-onset symptoms [1,2,3]. Early detection and accurate classification of ovarian masses are crucial for improving patient management and providing early treatment [4]. Borderline ovarian tumors (BOTs) present a diagnostic challenge due to their rarity, their non-specific and variable ultrasonographic characteristics, and their need for personalized treatment [5].

From a diagnostic perspective, BOTs are particularly problematic. Their ultrasonographic appearance is variable and often overlaps with that of both benign and malignant lesions, making preoperative classification difficult [6,7]. Correct identification is especially important in younger patients, where fertility preservation may be a priority [8]. Ultrasound imaging is the primary diagnostic tool for evaluating ovarian masses due to its accessibility, cost effectiveness, and non-invasive nature [9]. Diagnosing BOTs remains a significant challenge, even for experienced sonologists, who, while extremely accurate, have a slightly lower accuracy in detecting BOTs compared to benign and malignant tumors [10]. It can also be extremely challenging for general gynecologists who lack specialized training in ultrasonography [6]. In recent years, a shortage of ultrasound experts has been reported, which further limits access to high-level ultrasound assessment for many patients [11]. Moreover, the heavy workload of these experts contributes to the problem and makes them susceptible to human error. All the above explain the need for objective, automated diagnostic tools that would assist clinicians in accurately classifying ovarian tumors. Artificial intelligence and deep learning approaches have already shown remarkable performance in medical image analysis, including the classification of ovarian tumors [12,13]. Convolutional Neural Networks (CNNs) have been widely used for medical imaging applications due to their ability to extract complex features from images and facilitate diagnosis [11,14]. One of the main barriers to improving the performance of these models in low-incidence tumors such as BOTs is the difficulty in acquiring large and well-annotated data. To address this limitation, Generative Adversarial Networks (GANs) have been employed to generate synthetic ultrasound images, augmenting the training dataset with the goal of improving model performance [15,16,17].

We aimed to investigate whether the use of a Deep Convolutional GAN (DCGAN) could improve the performance of a deep learning classification model by augmenting the BOT sub-dataset. We developed a CNN-based three-class model that was trained with ultrasound images and enhanced by DCGAN synthetic images of BOTs to classify ovarian masses as benign, BOT, or malignant. This study aims to address class imbalance in a realistic clinical dataset obtained by a single center and evaluate the potential use of DCGANs to enhance decision support tools preoperatively.

## 2. Materials and Methods

### 2.1. Dataset and Data Processing

This retrospective study utilized ultrasound data retrieved from our institutional archive covering the period from 2011 to 2025. Eligible patients were women who presented with adnexal masses and underwent preoperative ultrasound examination and were subsequently treated surgically at our hospital. Exclusion criteria included pregnancy, age younger than 18 years, more than four months between ultrasound assessment and surgery, poor-quality or non-representative ultrasound images, and incomplete clinical or histopathological data. Four individual expert gynecologists chose 6 images per case, ensuring a comprehensive representation of the tumor characteristics. Ultrasound examinations were obtained by multiple available ultrasound systems from different manufacturers, including General Electric Healthcare, Athens, Greece (LOGIQ P6, Voluson S6, S8, E6, and E10) and Samsung, Athens, Greece (HS40), introducing variability in imaging conditions. The final dataset consisted of 3816 ultrasound images from 636 women with histopathologically confirmed ovarian masses, including 390 benign, 202 malignant, and 44 borderline ovarian tumors (BOTs). Among the BOT cases, histological subtypes included serous (*n* = 25), mucinous (*n* = 14), and seromucinous tumors (*n* = 5). Postoperative histopathology reports were used for the final classification of the ovarian masses in accordance with FIGO staging criteria [18]. The study protocol was approved by the Scientific Board of our institution (approval no. 30/28 June 2022), which waived the requirement for written informed consent due to the retrospective use of anonymized data.

### 2.2. Data Processing and Augmentation Using GANs

Raw ultrasound images are inherently susceptible to noise, which can severely obscure fine tissue details. To mitigate this, a median filter with a 3-by-3 kernel was applied to them, effectively smoothing granular noise. Following denoising, automated Region of Interest (ROI) cropping was utilized. The cropping algorithm incorporated thresholding, along with a binary mask of the ovarian mass, removing the surrounding pelvic anatomy without manual intervention. The masked ROIs were then extracted and resized to match the CNN input layer of the model. The input layer was also prepended by a flattened layer, as the original images were in RGB format (three color channels). Finally, normalization was performed to standardize the pixel intensity across the dataset, ensuring a mean of zero and a standard deviation of one.

**Figure 1:**
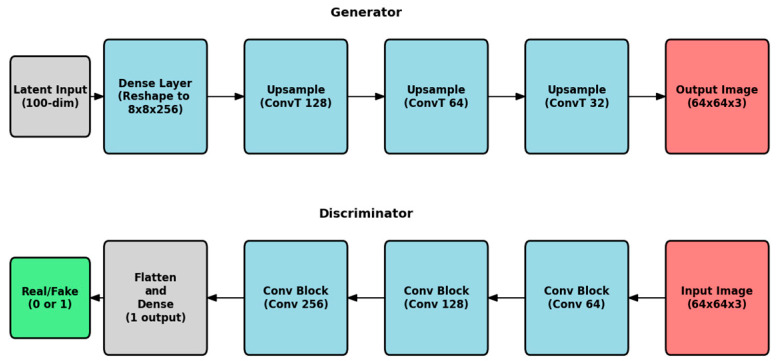
Top-level GAN architecture of both generator and discriminator.

As mentioned earlier, the scarcity of BOT ultrasound images severely limits the effectiveness of traditional data augmentation techniques. Geometric transformations (rotation, flipping, and cropping), color space transformations (brightness and contrast adjustment), or noise injection can all be utilized to enhance the size of the dataset [19]. However, as thoroughly assessed in previous research, the limited number of original images still leads to model overfitting [20]. Overfitting is already a prominent issue when building multi-class models, such as the one developed for this study. This was the main motivation for employing GANs to generate synthetic BOT ultrasound images [21]. GANs consist of two networks, a generator and a discriminator [22]. The generator takes a random noise vector as input and upsamples it to a generated image of the desired size. The discriminator takes images as input, both real and generated, downsizes them, and classifies them as real or artificial [23]. The GAN architecture used in this study was based on the DCGAN framework, which incorporates convolutional layers to enhance image generation [24]. The DCGAN architecture was used for both networks. Therefore, each of them incorporates three convolutional layers, with the generator and the discriminator having transposed layers, which are standard for the discriminator. Batch normalization and LeakyReLU activation are applied to all layers except for the input and output layers. The DCGAN was trained on the original BOT images, and once the generator produced realistic artificial images, these were added to the training dataset. Top-level GAN architecture can be seen in Figure 1.

The binary cross-entropy log loss function was used during the training process, essentially minimizing the discriminator’s ability to detect that the images built by the generator are artificial [25].

Mathematically, Binary Cross-Entropy (BCE) is defined as:

$$BCE=\;-\;\frac{1}{N}\sum_{i=1}^{N}\left[y_{i}\log\left(p_{i}\right)+\;\left(1-y_{i}\right)\log\left(1-p_{i}\right)\right]$$

- N is the number of observations;
- y_i_ is the actual binary label (0 or 1) of the ith observation;
- p_i_ is the predicted probability of the ith observation being in class 1

The dataset initially consisted of 3816 ultrasound images, collected from 636 women diagnosed with ovarian tumors. To mitigate the severe class imbalance between benign, malignant, and BOT images, which posed a challenge for training a robust CNN classifier, the Deep Convolutional GAN (DCGAN)-based model was employed to generate additional synthetic BOT images.

**Figure 2:**
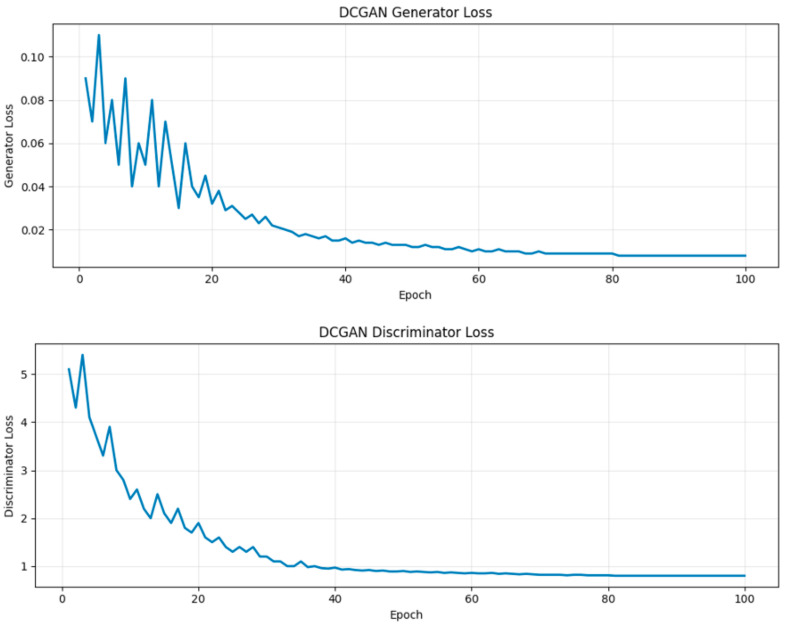
DCGAN training. Training stabilization in the DCGAN generator and discriminator after 32 epochs.

For the DCGANs, training stabilized and stopped after 32 epochs. Training stabilization can be seen for both the discriminator and generator DCGAN in Figure 2.

### 2.3. Model Development

**Figure 3:**
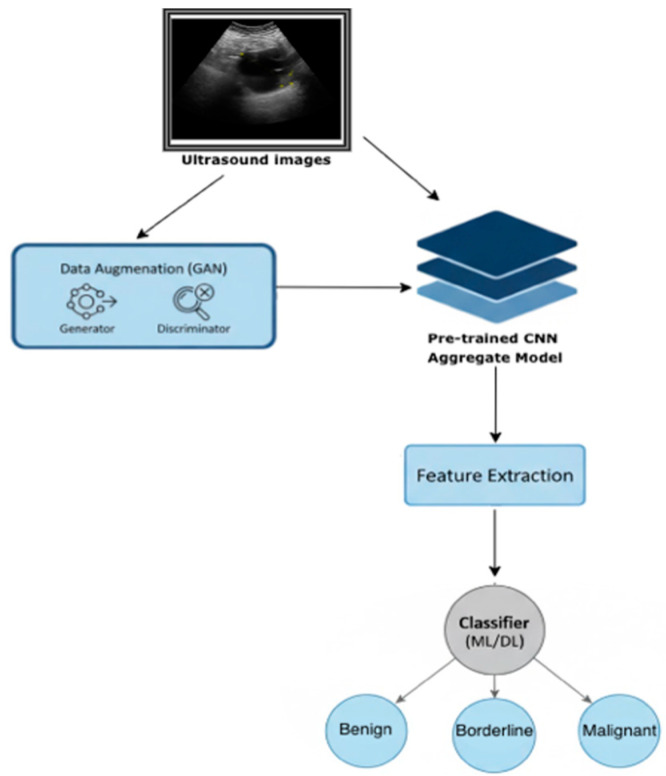
Flowchart of the model, using GANs for ovarian mass classification.

An important parameter to consider when designing GANs is the resolution of the generated images. High-resolution outputs (e.g., 256 × 256) introduce two main challenges. First, training a GAN at higher resolutions demands significantly greater computational power. While this requirement may seem trivial in many technical fields and industries, it is not always guaranteed in the medical domain, where resources can be limited. Moreover, medical experts may be reluctant to allocate substantial resources to technical infrastructure, as their priorities are often directed towards clinical needs. Second, the training process at high resolution suffers from instability and a heightened sensitivity to hyperparameter choices. Because the likelihood of the image distribution and the model distribution sharing supports in high-dimensional space is very low, mode collapse frequently occurs, leading to generated images with repetitive color and texture patterns. On the other hand, drastically reducing the resolution of synthetic images would undermine this study, as the goal is to use them for developing and training an ovarian tumor classification model. Therefore, a resolution of 64 × 64 was chosen as a sufficient compromise. In contrast to earlier binary classification framework, the current model was redesigned to address a three-class architecture to classify tumors into benign, malignant, and BOTs [12]. The final classifier for each image was essentially a weighted average of the three output probabilities as depicted in the flowchart (Figure 3). This approach was chosen to address the clinical challenge of differentiating BOTs from malignant tumors, which often share similar imaging characteristics [26]. All model training and evaluation were performed using fixed random seeds to ensure reproducibility.

## 3. Results

### 3.1. Image Generation

**Figure 4:**
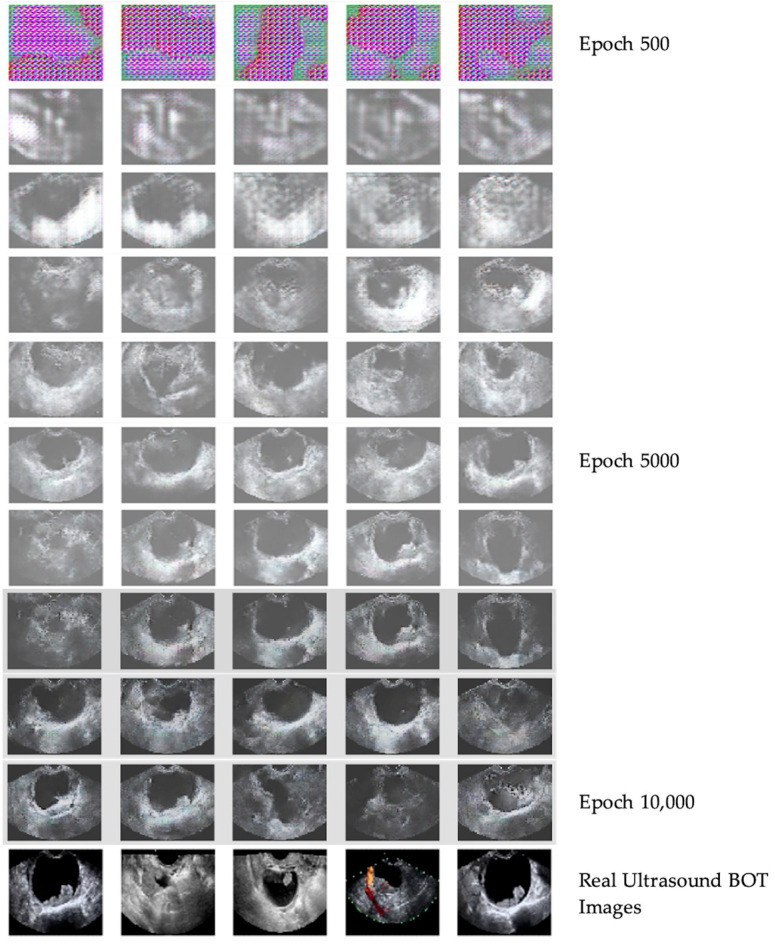
GAN training process. GAN-generated images of BOTs during different epochs versus ultrasound-obtained images of BOTs at 64 × 64 pixel resolution.

A total of 2000 GAN-generated BOT images were incorporated into the training dataset. After augmentation, the final training set consisted of 2340 benign, 1212 malignant, and 2264 BOT images, resulting in a more balanced class distribution. Synthetic images were used exclusively for training. The training process and GAN-generated images during different epochs, in comparison with real ultrasound-obtained BOT images, can be seen in Figure 4. In the first 4000 epochs, the general structure of artificial ultrasound images was formed. After 5000 epochs, key BOT imaging features were gradually introduced into the synthetic images. Multilocular cysts, thin septations, and papillary projections were created by GANs progressively until 10,000 epochs, where the synthetic images were finalized.

A custom-tailored assessment technique was applied to evaluate the quality of the GAN-generated images. Quantitative GAN evaluation metrics such as FID or IS were not used since they are optimized for natural images and may not reflect the diagnostic realism in ultrasound imaging. Instead, experienced sonologists visually assessed and inspected generated images for artifacts that could bias CNN training. Each GAN-generated image was qualitatively evaluated by four experienced sonologists, using a 5-point Likert-type scale to measure perceived realism [27]. The realism score was 4.3 ± 0.6, indicating high visual plausibility.

### 3.2. Model Training

The classification model was implemented by combining different neural networks (VGG16, ResNet50, and InceptionNetV3) [12]. Individual network outputs were combined through a weighted fusion strategy to generate the final class prediction [12]. The final model was fine-tuned on the augmented dataset, which included both original and GAN-generated images. A 70% training, 15% validation, and 15% test split was used on the dataset. However, the real images were flagged, and evaluation metrics were only calculated over real and not synthetic data. All dataset splits (training, validation, and test sets) were performed strictly at the patient level, ensuring that images from the same patient were never distributed across different subsets. Cross-validation was also employed (k-fold), and the results were averaged out over 5 different folds. Early stopping was used to interrupt the training process based on validation loss.

### 3.3. Model Performance

All analyses were performed using Python 3.10 with NumPy 1.26, SciPy 1.11, pandas 2.1, and scikit-learn 1.4. Model performance was evaluated using sensitivity, specificity, F1-score, and area under the curve (AUC). Cross-validation was performed to reduce optimism bias and provide a stable internal estimate of model performance. Ninety-five percent confidence intervals (95% CIs) were estimated using a non-parametric bootstrap method with 1000 resamples of the test set. For each resample, the full set of performance metrics was recomputed, and the 2.5th and 97.5th percentiles of the resulting distributions defined the confidence intervals. Confidence intervals represent variability across bootstrap resamples of the independent test set and should be interpreted as internal uncertainty estimates rather than population-level inference. The F1-score is the harmonic mean of precision and sensitivity, calculated from this formula:

$$F1\text{-}score=\;2\times \frac{Precision\;\times \;Sensitivity}{Precision\;+\;Sensitivity}$$

Unlike the simple arithmetic mean, the harmonic mean penalizes models with a large imbalance between precision and sensitivity. Because clinical decision making requires balancing missed malignancies against unnecessary surgery, no single metric fully captures the diagnostic performance. Sensitivity reflects safety by minimizing false negative cases, specificity can minimize overtreatment, the F1-score summarizes performance under class imbalance, and the AUC captures threshold-independent discrimination.

**Figure 5:**
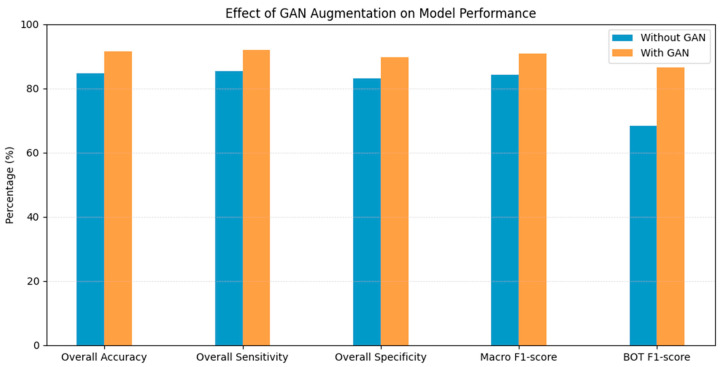
Effect of GAN-generated images on model performance. Comparison of performance metrics of the final model with and without GAN augmentation.

The high F1-score indicates that the classifier achieves a favorable balance between sensitivity and precision, reflecting effective identification of positive cases while minimizing false alarms. On trial runs without GAN augmentation, the BOT class F1-score for the same three-class model was measured as 68.4%. With the employment of DCGANs, this improved to 86.5%, showing a 25.8% relative increase. After using DCGANs, the model achieved a training accuracy of 96.8%, validation accuracy of 92.3%, and test accuracy of 91.5%, demonstrating strong generalization performance. Similarly, the overall accuracy increased from 84.7% in the test set for the baseline model to 91.5% in the DCGAN augmentation model (Figure 5).

These findings underline the importance of GAN-based augmentation in improving the classification of BOTs. The macro-averaged specificity, sensitivity, and F1-score for the final model after training with the DCGAN-generated BOTs images were 89.7%, 92.1%, and 90.8%, respectively. The mean AUC across all classes was 0.94, indicating strong overall discriminative performance. The sensitivity, specificity, F1-score, and AUC of the model for each class can be seen in Table 1.

**Figure 6:**
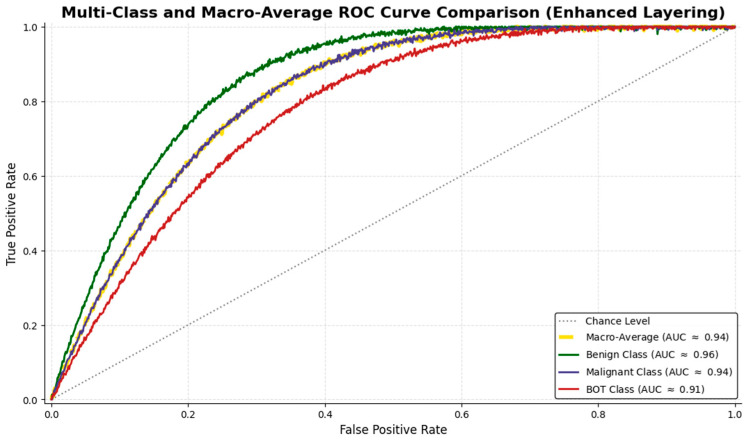
ROC curve comparison for benign, malignant, and BOT of the final model using GAN augmentation techniques.

Receiver Operating Characteristic (ROC) curve analysis further documented robust class separation, with AUC values of 0.96 for benign, 0.94 for malignant, and 0.91 for BOT images (Figure 6).

**Figure 7:**
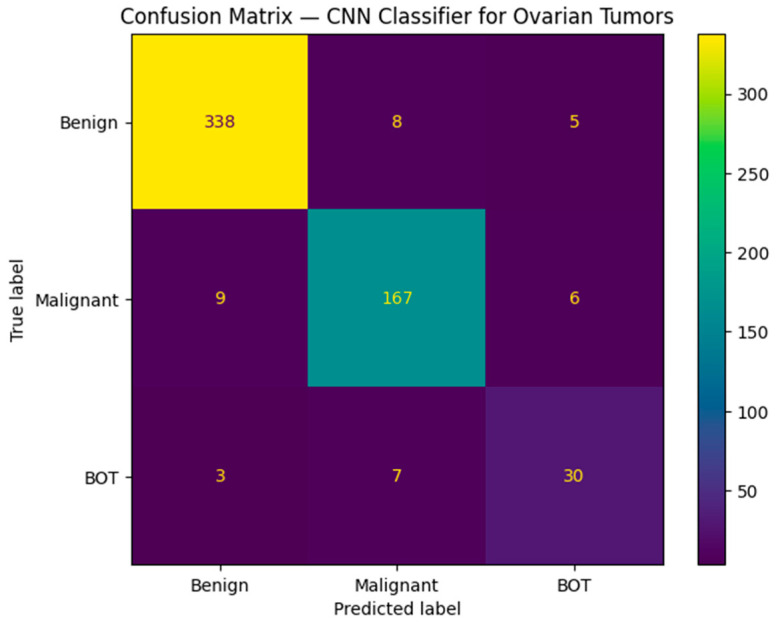
Confusion matrix of the final model between the three classes.

The confusion matrix shows that the largest source of residual misclassification involved BOT images being predicted as malignant, reflecting their intermediate and often malignant sonographic morphology due to solid areas, such as papillary projections or septations (Figure 7). This pattern mirrors real-world diagnostic challenges encountered in clinical practice and emphasizes the inherent complexity of BOT classification.

## 4. Discussion

Correct preoperative discrimination between benign, malignant, and BOTs is essential for choosing the appropriate patient management approach [28]. BOTs, in particular, are a very important entity due to their low potential for metastasis but high risk of recurrence [29]. They are treated surgically, but the type of surgery and the management strategy can vary based on factors such as the patient’s age and fertility preservation [8]. On ultrasound examination, BOTs can exhibit complex features, sometimes similar to both benign and malignant tumors, making their diagnosis challenging [6,7]. Although ultrasound is the reference imaging modality for adnexal mass evaluation, subjective assessment can lead to substantial diagnostic variability [9,30,31]. Several studies have documented the difficulty of identifying BOTs using traditional methods, even by experienced sonologists [6,11,32,33]. In settings without access to medical centers with experts and qualified sonologists, this problem may be even more pronounced.

As a result, there is growing interest in the development of automated ultrasound image analysis systems. Deep learning modules are often utilized to complete the task of discriminating ovarian ultrasound images, but when BOTs are included as a separate entity, the sensitivity, specificity, and overall accuracy drop [34]. In the paper by Christiansen et al., we can also see a significant lower model sensitivity (69%) for BOTs compared to benign and malignant tumors, which is consistent with the results of our model before employing the DCGANs [11]. Thus, the need for objective diagnostic systems to help clinicians accurately distinguish between benign, malignant, and borderline tumors is critical. The high cost, effort, and time required to obtain and annotate medical ultrasound images by already overworked clinicians seem to be the main setbacks to advancing and training convolutional networks efficiently. One of the most substantial obstacles encountered in medical image analysis, particularly in rare tumors such as BOTs, is the imbalance in dataset distribution. The limited number of BOT cases can severely affect the model’s ability to generalize well, leading to overfitting and poor classification performance [35]. To address these limitations, data augmentation techniques such as rotation, flipping, and zooming are often employed. Although these can be effective for certain simple tasks, they are not enough to address the already technically demanding classification of rare tumors with varied ultrasonographic appearance [19]. In recent years, the use of Generative Networks has been explored in the field of medical imaging due to their remarkable ability to generate images and enrich imaging datasets of under-represented classes [15,36]. Previous studies have explored the use of synthetic image generation across a range of medical imaging modalities, including breast ultrasound, cardiac cine-MRI, and liver CT, with promising outcomes [16,21,37,38,39]. DCGANs have also been employed for ovarian tumor classification with the goal of augmenting limited ultrasound image datasets, demonstrating favorable results by improving the accuracy and performance of CNN models [19]. A previously validated binary classifier was restructured to develop a three-class discriminator for classifying ovarian masses in three categories: benign, malignant, and BOTs [12]. To augment the BOT dataset, we employed DCGANs to address this size imbalance between the three categories. We evaluated the efficacy of GANs in generating realistic medical images and their downstream utility in clinical tasks. The three-class model achieved high sensitivity and specificity, with an F1-score of 86.5% for BOTs with the use of DCGANs compared to 68.4% without them. This significant improvement in the performance of the model indicates the potential use of DCGANs in clinical practice to aid the identification of rare tumors. The successful application of CNNs and DCGANs in ovarian tumor classification could have remarkable implications in reducing human error and facilitating the diagnosis of not only BOTs but also other rare ovarian tumors, such as solid benign masses. Having access to a diagnostic tool that can help overcome the challenges presented by these types of ovarian pathologies could eventually reduce the number of radical surgeries for women wishing to maintain their fertility, or it could even lead to an earlier diagnosis of BOTs for tumors with more subtle characteristics. Integrating CNNs and DCGANs into existing clinical workflows can be a valuable tool for clinicians in their everyday clinical practice [40]. The proposed model is intended as a decision support or second-reader tool. It could serve as a first-step classification tool in resource-limited settings where access to expert sonologists is unavailable. Its potential applications include triage of complex cases for referral to tertiary oncology centers and guidance for non-expert gynecologists. While real-time inference was not evaluated, the architecture is compatible with near-real-time deployment. While the results of our study are promising, the next and more crucial step is to train the models with data acquired from other centers and different populations to test their performance in other clinical settings with diverse ultrasound imaging conditions. Moreover, the integration of these models into everyday clinical practice is needed so that continuous feedback and model refinement will ensure their optimization.

While this study demonstrates promising results, several limitations must be acknowledged, such as the initial number of BOTs, which remains relatively small. As stated before, BOTs are rare, and despite extensive data collection, only 44 histopathologically confirmed BOT cases were available. This reflects real-world clinical prevalence and represents the main limitation of the study. While DCGAN-based augmentation improves model learning under these constraints, synthetic images cannot replace the full ultrasonographic morphological features of real clinical data. Although the number of BOT cases was limited, the use of patient-level splitting and cross-validation provides a careful and transparent internal assessment of model performance. The reported metrics therefore reflect reliable performance within the studied population, while acknowledging that external validation is required to confirm the model’s generalizability.

Another limitation is that the data used for model training and testing are derived from only one center, which may introduce center-specific biases related to patient population, operator expertise, ultrasound protocols, and institutional practices. Although images were acquired using six different ultrasound systems and obtained by different sonologists, this does not substitute for true multi-center validation. This represents technical heterogeneity within one institution and does not substitute for external validation; therefore, generalizability to other clinical settings remains crucial. A more efficient training process and accurate evaluation of the model can be achieved by combining data from multiple centers and creating a larger dataset. External validation using data from additional centers and populations is a critical next step and will be the focus of future work.

All patients included in the study were adults; therefore, the findings of this study should not be generalized to pediatric or adolescent populations. Since the proposed model has not been tested for women younger than 18 years, it should be used with caution outside adult populations. In younger populations, certain lesion types—particularly germ cell tumors—are more prevalent than in adults, leading to differences in diagnostic pathways and treatment priorities, especially with respect to fertility preservation [41,42,43]. The clinical relevance of accurate preoperative classification of ovarian masses, however, remains and extends across women’s reproductive lifespan. Fertility preservation management is increasingly important for many women, including those pursuing assisted reproductive technologies later in life. Future studies including broader and more diverse patient cohorts will be necessary to further evaluate model performance across different clinical and reproductive scenarios. Finally, although the 64 × 64 resolution limits the explicit visualization of fine morphological details, such as septations and papillary projections, the synthetic images were intended to support CNN feature learning rather than direct clinical interpretation. Even at this scale, the classifier showed improved performance, indicating that relevant ultrasound patterns were preserved. Nevertheless, future studies should explore higher-resolution or multi-scale GAN architectures on larger datasets.

## 5. Conclusions

In conclusion, this study highlights the significant potential of combining CNNs and DCGANs for the classification of ovarian tumors, including the challenging task of accurately identifying BOTs. By using DCGANs to augment the ultrasound image dataset, we have shown that it is possible to overcome the challenges created by less common types of ovarian tumors, such as BOTs, which are often difficult to diagnose even by expert sonologists. Our study supports the use of Convolutional Neural Networks in medical ultrasound imaging, offering promising results for the improvement of preoperative diagnostic accuracy regarding ovarian masses and facilitating decision making for patients and clinicians. Future work should focus on exploring more advanced GAN architectures or hybrid approaches, refining the model with more diverse datasets, and incorporating data from multiple centers.

## Figures and Tables

**Table 1:** Model performance metrics.

Histopathology	Sensitivity (%) (95% CI)	Specificity (%) (95% CI)	F1-Score (%)	AUC (95% CI)
*Benign*	96.4 (91–98.2)	94.2 (93.2–95.5)	95.3	0.96 (0.91–0.98)
*BOT*	88.2 (83.9–92.3)	85.1 (79.9–88.1)	86.5	0.91 (0.85–0.94)
*Malignant*	91.8 (90.6–96.3)	89.7 (84.8–92.5)	90.6	0.94 (0.89–0.97)

Per-class performance results for the final model summary. AUC: area under the curve; CI: confidence interval.

## Data Availability

The dataset used in our study is not publicly available due to patients’ privacy and research permissions, but the data and results are available upon reasonable request from the corresponding author.
